# Can genetics guide exercise prescriptions in osteoarthritis?

**DOI:** 10.3389/fresc.2022.930421

**Published:** 2022-07-29

**Authors:** Osvaldo Espin-Garcia, Madhu Baghel, Navraj Brar, Jackie L. Whittaker, Shabana Amanda Ali

**Affiliations:** ^1^Department of Biostatistics, Princess Margaret Cancer Centre and Schroeder Arthritis Institute, University Health Network, Toronto, ON, Canada; ^2^Division of Biostatistics, Dalla Lana School of Public Health and Department of Statistical Sciences, University of Toronto, Toronto, ON, Canada; ^3^Bone and Joint Center, Department of Orthopaedic Surgery, Henry Ford Health, Detroit, MI, United States; ^4^Faculty of Kinesiology and Physical Education, University of Toronto, Toronto, ON, Canada; ^5^Department of Physical Therapy, Faculty of Medicine, University of British Columbia, Vancouver, BC, Canada; ^6^Arthritis Research Canada, Vancouver, BC, Canada; ^7^Center for Molecular Medicine and Genetics, School of Medicine, Wayne State University, Detroit, MI, United States; ^8^Department of Physiology, College of Human Medicine, Michigan State University, East Lansing, MI, United States

**Keywords:** disease management, epigenetics, genomics, physical activity, precision medicine, intervention

## Abstract

Osteoarthritis (OA) is the most common form of arthritis and has a multifactorial etiology. Current management for OA focuses on minimizing pain and functional loss, typically involving pharmacological, physical, psychosocial, and mind-body interventions. However, there remain challenges in determining which patients will benefit most from which interventions. Although exercise-based interventions are recommended as first-line treatments and are known to be beneficial for managing both the disease and illness of OA, the optimal exercise “prescription” is unknown, due in part to our limited understanding of the precise mechanisms underlying its action. Here we present our perspective on the potential role of genetics in guiding exercise prescription for persons with OA. We describe key publications in the areas of exercise and OA, genetics and OA, and exercise and genetics, and point to a paucity of knowledge at the intersection of exercise, genetics, and OA. We suggest there is emerging evidence to support the use of genetics and epigenetics to explain the beneficial effects of exercise for OA. We identify missing links in the existing research relating to exercise, genetics, and OA, and highlight epigenetics as a promising mechanism through which environmental exposures such as exercise may impact OA outcomes. We anticipate future studies will improve our understanding of how genetic and epigenetic factors mediate exercise-based interventions to support implementation and ultimately improve OA patient care.

## Introduction

Osteoarthritis (OA) is a chronic joint condition with increasing prevalence worldwide. Currently, there is no cure for OA nor approved disease-modifying OA drugs (DMOADs). Clinical practice guidelines consistently recommend exercise as an effective first-line treatment for knee, hip, hand, and polyarticular (or generalized) OA (1, 2). Despite knowing the benefits of exercise for managing OA for over a decade (3), implementation of exercise interventions remains a challenge. Among the hurdles is a lack of clarity about the precise mechanisms through which exercise improves OA outcomes, including whether specific types of exercise are better suited to specific OA patient populations. Biomechanical hypotheses have been explored at molecular, cellular, tissue, organ, and system levels in OA (4). Yet exercise can have different effects on outcomes of OA *disease* (i.e., mitigating structural changes to the joint) and OA *illness* (i.e., reducing symptoms experienced by the patient) (5), adding complexity to exercise prescription for OA patients where discordance between structure and symptoms may exist.

The emerging field of molecular exercise physiology suggests genetics may be critical for understanding potential mediators of the effect of exercise on health and disease (6). Both exercise (7) and OA (8) are associated with genetic variants, with new risk loci continuing to emerge depending on the phenotype definition. For OA, these genetic data explain only a fraction of the phenotypic variation observed, suggesting other factors are also at play. Epigenetic factors—DNA methylation, histone modification, and non-coding RNAs—play important roles in regulating gene expression and are highly responsive to environmental variables (9). As a key environmental factor, exercise may induce epigenetic changes that can mitigate OA, and these effects may be context-dependent (e.g., patient-specific). Therefore, from our perspective, if genetics and epigenetics can mediate the effects of exercise on OA outcomes, they can potentially be used to guide OA exercise interventions. However, there is a paucity of existing research at the intersection of genetics, exercise, and OA.

### Exercise and OA

A meta-analysis published in 2019 with, “No new trials on exercise in knee OA,” in the title concluded based on studies dating back to 1992 that exercise is clearly an effective intervention for reducing pain in patients with knee OA compared to no or minimal treatment (3). Similarly, both the *American College of Rheumatology/Arthritis Foundation guideline for the management of osteoarthritis of the hand, hip, and knee* (1), and the *Osteoarthritis Research Society International guidelines for the non-surgical management of knee, hip, and polyarticular osteoarthritis* (2), among other OA guidelines, strongly recommend exercise, including aerobic, resistance, and neuromuscular exercises. Different exercise types confer different physiological adaptations that can benefit OA management (Table 1). However, there is currently insufficient evidence to guide the choice of exercise type and the “dose” (intensity, frequency, duration). Information about when in the disease trajectory exercise should be undertaken, and which patients do best, are also limited. These gaps in knowledge are in part due to a lack of understanding precisely how exercise improves OA outcomes (10).

**Table 1:** Overview of exercise interventions for OA.

**Exercise type**	**Definition**	**Examples**	**Benefits for OA** ^*^
Aerobic exercise	• Exercise that uses large muscle groups, can be maintained continuously, and are rhythmic in nature (11). • Moderate and vigorous aerobic exercise increase breathing and heart rate, are perceived to be “hard” and cause perspiration. • Exercise that relies on energy produced by oxygen.	• Moderate: brisk walking or bicycling, swimming • Vigorous: jogging, aerobic dance or bicycling uphill (produces large increases in breathing or heart rate)	• Improves mobility (12) • Reduces pain (12) • Improves muscle capacity (mass, strength, power and/or endurance) (13) • MVPA benefits cardiovascular health (14) • MVPA assists in weight management (15) • MVPA reduces risk of comorbidities (16) • Benefits skeletal health (17)
Resistance-based exercise	• Exercise that causes the muscles to contract against an external resistance with the expectation of increasing muscle mass (hypertrophy), strength, power, and/or endurance (18).	• External resistance can come from weight machines, dumbbells, kettle balls, exercise tubing, body weight etc.	• Improves mobility (12) • Reduces pain (12) • Improves muscle capacity (mass, strength, power and/or endurance) (13) • Benefits skeletal health (17) • Reduces risk of injury and falls (19)
Neuromuscular control exercise	• Exercise that causes muscles to contract in a coordinated manner to control movement (20). • Exercise that incorporates functional movements involving multiple joints and muscle groups (20).	• Balancing on one or two legs • Planting and pivoting • Transferring body weight • Stepping up or down stairs • Squatting	• Improves mobility (12, 20) • Reduces pain (12, 20) • Improves movement confidence (21, 22) • Improves balance (21, 22) • Improves movement efficiency (21, 22) • Reduces risk of injury and falls (19)

* Exercise categories can have overlapping benefits.

MVPA, moderate to vigorous physical activity.

Since exercise induces perturbations to the mechanical environment of tissues in the joint, exercise and mechanobiology are intimately related (4). Mechanobiological adaptations can impact tissue structure (e.g., geometry), function (e.g., material property), and signaling (e.g., mechanosensitive genes), and therefore represent potential mechanisms underlying the benefits of exercise (23). It is well established that cells are sensitive to mechanical loading and can respond with alterations in diverse functions, including cell proliferation, production of soluble factors, and expression of extracellular matrix genes/proteins. There are specific cellular components that act as sensors of mechanical load, such as the cytoskeleton, integrins, G proteins, kinases, and stretch-activated ion channels (24). These “sensors” transmit the type and magnitude of the forces experienced by cells from the extracellular milieu. In turn, this can activate intracellular signaling cascades, alter gene expression profiles, and ultimately modify tissue properties (25). Even with this understanding of the link between exercise and mechanobiology, we have yet to decipher which exercises will most benefit the individual OA patient.

Despite insurmountable evidence for the effectiveness of exercise in OA management, and progress made implementing education and exercise-based interventions [e.g., the Good Life with osteoArthritis from Denmark (GLA:D^®^) program, now in over 7 countries globally (26)], there remains hesitation about the benefits of exercise for OA which is a barrier to its uptake by patients and health care professionals (27). Although any movement (i.e., physical activity) is better than none when it comes to OA, it is often counterintuitive for patients who are experiencing movement-related pain to see exercise as beneficial. Moreover, the evidence from exercise trials is limited by the inability to have an appropriate control arm since a true placebo group is not possible (i.e., participants know whether they are exercising). One way to overcome this “control condition” challenge may be to design more pragmatic exercise trials (28), including comparison of different exercise types, and variations in intensity, frequency, and duration of exercise. It would also be beneficial to have clearly defined hypotheses about the mechanisms through which exercise improves OA outcomes to support more tailored exercise interventions in future studies.

### Genetics and OA

In the past decade, genome-wide association studies (GWAS) have uncovered dozens of novel OA genetic risk loci and validated previously associated genomic regions. The specific OA loci identified and current state of OA genetics have been reviewed by Aubourg et al. in a recent article (8). Indeed, with heritability estimates of radiographic OA of 50% or more (29), the role of genetics in OA has been long recognized. However, OA is a complex condition, and phenotyping is challenging in part due to the dichotomy between OA *disease* and *illness* (5). The largest OA GWA study to date performed analyses on 11 OA phenotypes comprising 826,690 individuals spanning across 9 populations in 13 cohorts (30). These phenotypes represent presence/absence of OA across joints, as well as joint replacement status. Boer and colleagues identified 223 independent genetic risk loci across phenotypes including 84 variants that had not been previously associated with OA. These novel findings pinpoint risk loci related to neuropathology, sex-specific effects, and early OA (30). Moreover, the authors found evidence of genetic correlation between OA and pain phenotypes including sciatica, fibromyalgia, and headaches. Such a carefully designed large-scale GWA study increases our understanding of the genetic etiology of OA.

Despite these new discoveries and their potential to guide DMOAD development (30), multiple opportunities remain. For example, most studies focus on identifying variants associated with OA risk. However, understanding OA trajectories over time may bring additional biological insights, thus, progression phenotypes offer a complementary approach that deserves further investigation (31). Alternatively, since genetics constitute only a fraction of OA etiology, exploring environmental triggers and modulators (e.g., exercise) will further increase our understanding of OA pathophysiology. In this context, it is crucial to investigate the interaction between genetics and epigenetics. Most of the identified risk loci are in non-coding genomic regions and may increase disease risk by modulating the expression of target genes while correlating with epigenetic mediators, representing a potential mechanism linking known risk factors of OA with its progression (32). On this note, non-coding RNAs (e.g., microRNAs) and their interactions present themselves as promising biomarkers, mediators of pathogenic mechanisms, and potential therapeutic targets for OA (9).

### Exercise and genetics

A substantial body of research in the field of *sports and exercise genetics* has improved our understanding about the biological underpinnings of exercise, including the role of adaptation and the influence of DNA variability (33–35). This seminal work has established a genetic basis for aerobic performance measures (e.g., maximal oxygen uptake) via heritability analysis in family studies (34), suggesting a link between genetics and aerobic capacity. Although these aerobic performance measures are the gold standard for measuring cardiorespiratory fitness (a key aspect of physical fitness and physical activity), their collection is often impractical, costly, and can pose ethical concerns as they require maximal exertion from the participants. Thus, large-scale studies have instead relied on self-reported physical activity phenotypes which may be subject to perception, desirability, or recall biases (36–38). To circumvent these limitations and biases there is a need to objectively measure exercise and the resulting forces on local tissues (39). Fortunately, wearable devices including accelerometers, pedometers, and biosensors are increasingly available and provide an alternative to self-report (40, 41).

A recent systematic review identifying genetic variants associated with physical activity or sedentary behavior highlights 54 studies spanning the last three decades (7). Of these, six GWAS identified 10 single nucleotide polymorphisms (SNPs) at a genome-wide significance level, while the remaining 48 studies used a candidate gene approach, and collectively identified 30 different genes. However, the findings were vastly inconsistent across studies mainly due to the variability in phenotype definition, sample size, study population, and study design (7). Notably, among the six GWAS, one high-quality study leveraging accelerometer data from 91,105 participants from the UK Biobank is consistent with previous findings on the role played by the central nervous system in activity behaviors (42). Moreover, an updated analysis of the UK Biobank accelerometry sub-study further suggests that the blood and immune system can be associated with exercise (43).

Several limitations arise in the existing evidence on the genetics of physical activity or movement. First, investigated phenotypes—even objectively measured ones—aggregate information across different types of physical activities (e.g., leisure and work). Moreover, it is unclear how different exercise types may influence the identified associations. Second, there is a lack of translation in using current findings to inform intervention guidelines and prevention strategies for physical activity and beyond. Moving toward this goal, recent work identified associations between a polygenic score of physical activity and multiple complex diseases (44). Finally, existing genomic data have been limitedly integrated with other -omics technologies that could reflect epigenetic changes. Leveraging such integrations may increase our ability to explain the “missing heritability” of physical activity by unmasking potential gene-environment interactions.

### Exercise, genetics, OA

Though there is research exploring exercise and OA, genetics and OA, and exercise and genetics, there are few studies connecting exercise, genetics, and OA. As one possible link, emerging studies are focusing on epigenetics, including non-coding RNAs and DNA methylation. A report published in 2021 found changes in expression of the long non-coding RNA (lncRNA) H19 in cartilage following high- and moderate-intensity treadmill running which promoted or mitigated, respectively, knee joint damage in a post-traumatic OA mouse model. The authors suggest lncRNA H19 may be interacting with microRNAs to influence osteogenic differentiation in response to mechanical stress (i.e., exercise) (45). In patients undergoing knee replacement, DNA methylation rates at CpG1 in the pyruvate dehydrogenase kinase 4 (PDK4) gene were explored in skeletal muscle and peripheral blood before and 5 months after resistance training and aerobic exercise. Though no differences in methylation of *PDK4* were found with exercise, potentially due to the small sample size (*N* = 5), there was a significant correlation in methylation rates between the tissue and blood samples. This suggests epigenetic factors can be measured in clinical settings using minimally invasive liquid biopsies as a surrogate for muscle tissues (46). While both studies investigate the premise that epigenetic factors are effectors of exercise in OA, an outstanding question remains as to whether genetic and epigenetic factors can be used to guide exercise interventions in OA care.

## Discussion

Contrary to widespread myths that exercise exacerbates OA, there is an abundance of evidence demonstrating the beneficial effects of exercise for OA (3). However, there is limited evidence as to precisely *how* exercise improves OA outcomes, contributing to challenges for widespread implementation of exercise as an intervention for OA. Since genetic factors explain only about half of the heritability observed in OA, it is likely that environmental factors such as exercise also play a role, the effects of which may be mediated by epigenetic factors (32). With advances in the field of molecular exercise physiology, including improved technology for objectively measuring physical activity, we are gaining a better understanding of the biological mechanisms underlying exercise (6). Despite this, few studies to date directly investigate the effects of exercise and genetics on OA outcomes. Among the examples we identified, non-coding RNAs and DNA methylation are two epigenetic mechanisms that have been proposed as potential effectors (45, 46). This suggests further exploring the intersection of exercise, genetics, and OA has the potential to guide delivery of evidence-based interventions to improve OA care.

**Figure 1:**
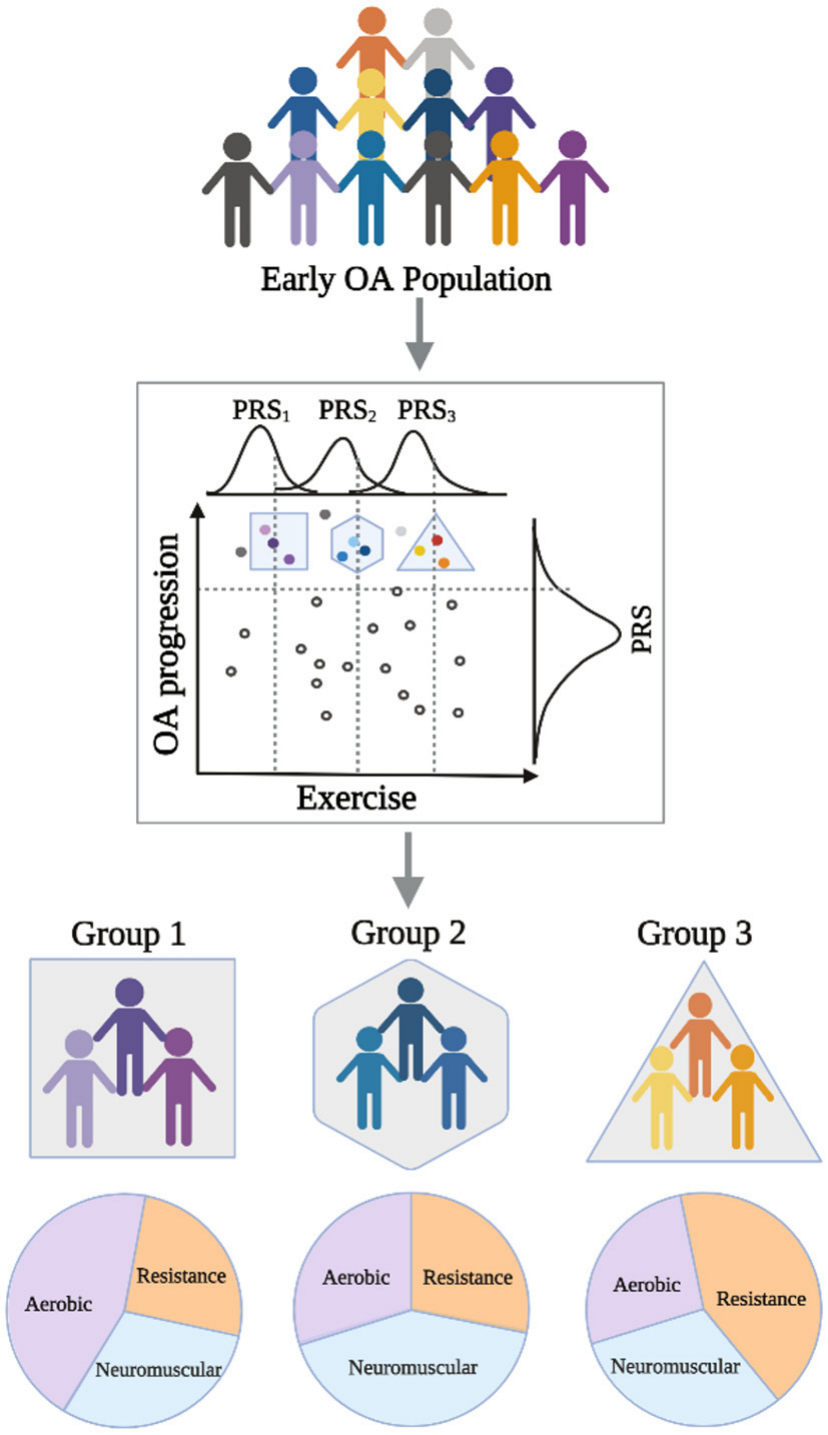
Schematic diagram showing applications of polygenic risk scores (PRS) in an early OA population to guide prescription of specific exercise interventions to improve outcomes.

Given the current state of the field, it is our perspective that we are approaching a point where efforts in genetic and epigenetic data collection in conjunction with detailed physical activity tracking can be leveraged to guide exercise interventions in OA. To this end, the construction of polygenic scores on OA progression and response to exercise types can serve to tailor interventions for early OA populations or at-risk populations by identifying subjects with an increased propensity to benefit from specific exercise combinations (Figure 1). These polygenic scores can be further informed by transcriptomic data, which have demonstrated improvements in their portability across ancestries (47), and potentially across more specific OA sub-populations (e.g., early OA). However, drawing lessons from ongoing investigations for cognitive function, it remains unclear how changes in genetic makeup may lead to better outcomes from different exercise types, and how gene-gene interactions play a role in exercise efficacy (48). One potential physiological mechanism through which exercise may benefit OA is mitigation of sarcopenia—the loss of muscle mass—since it has already been linked to genetic and epigenetic factors as well as complex diseases including OA (49–51). There are many other plausible hypotheses (e.g., mitigation of poor diet) and hypotheses yet to be formulated that are expected to emerge from and be tested in unbiased analyses of large-scale OA datasets.

We have identified three areas of opportunity to help realize the potential of the unprecedented amounts of data available to inform precision medicine in exercise interventions for OA. First, integrating data from wearable technologies with detailed physical activity self-reports is needed to distinguish among exercise types and activity levels. For instance, identifying human chronotypes—behavioral manifestations of underlying circadian rhythms of multiple physical processes—among early OA or at-risk populations can provide a framework to explain some heterogeneity in OA by differentiating activity profiles (52). Second, conducting gene-by-exercise interaction studies in large cohorts is needed to identify candidate loci that, in combination with epigenetics, will help in elucidating molecular mechanisms of exercise in human health and disease (e.g., OA progression). While resources like the UK Biobank have demonstrated great potential for complex diseases (53, 54), there is a need to perform these investigations in more diverse populations, especially as the risk of OA appears larger in individuals with African ancestry (55). Third, leveraging existing efforts in well-characterized populations is needed to discover and validate findings from one and two above. We expect initiatives like the *Grand Challenge Competition to Predict In Vivo Knee Loads* (56) and *All of Us* (57) will aid in catalyzing these investigations. As additional examples, the Athlome Project Consortium (58) and the Osteoarthritis Initiative (59) are two unique resources with available data that can be used to refine our understanding of the role of exercise in OA.

With the ultimate goal of achieving a precision medicine approach to OA care, where tailored interventions are delivered to the right patients at the right time to improve outcomes, genetics and environment are two critical variables. Here we focus on exercise as a key environmental factor shown to impact OA outcomes. A report published in 2021 found diet and exercise interventions, alone or together, had different effects on OA outcomes (e.g., pain) for different subgroups of participants (60). While baseline characteristics such as weight could explain the variation in responses, so too could differences in genetic or epigenetic factors. As more well-designed, large-scale, high-throughput analyses are conducted for OA, it can be expected that key genetic and epigenetic factors will be prioritized based on their association with desired responses to interventions, including exercise (61). These factors can be translated for use in clinical settings, where a visit to a doctor may involve a blood draw and analysis, the results of which are used to tailor a specific exercise prescription to the specific patient, much like polygenic scores are used to tailor chemotherapy regimens for cancer patients (62). The field is now poised to leverage genetics and epigenetics to guide exercise interventions in OA.

## Data availability statement

The original contributions presented in the study are included in the article, further inquiries can be directed to the corresponding authors.

## Author contributions

OE-G and SAA contributed to conception and design of the manuscript, and wrote the first draft. MB and NB prepared data for visualization and presentation. JW contributed to shaping the research question. All authors contributed to manuscript revision, read, and approved the submitted version.

## Funding

Funding to support this publication was provided to SAA by Henry Ford Health.

## Conflict of interest

The authors declare that the research was conducted in the absence of any commercial or financial relationships that could be construed as a potential conflict of interest.

## Publisher's note

All claims expressed in this article are solely those of the authors and do not necessarily represent those of their affiliated organizations, or those of the publisher, the editors and the reviewers. Any product that may be evaluated in this article, or claim that may be made by its manufacturer, is not guaranteed or endorsed by the publisher.
